# The photoprotective properties of α-tocopherol phosphate against long-wave UVA1 (385 nm) radiation in keratinocytes in vitro

**DOI:** 10.1038/s41598-021-01299-y

**Published:** 2021-11-17

**Authors:** M. M. Saleh, K. P. Lawrence, S. A. Jones, A. R. Young

**Affiliations:** 1 Department of Pharmaceutics and Pharmaceutical Technology, School of Pharmacy, The University of Jordan, Amman, 11942 Jordan; 2 St John’s Institute of Dermatology, King’s College London, London, SE1 9RT UK; 3 Institute of Pharmaceutical Science, Faculty of Life Sciences and Medicine, School of Cancer and Pharmaceutical Sciences, King’s College London, Franklin-Wilkins Building, 150 Stamford Street, London, SE1 9NH UK

**Keywords:** Skin cancer, Nanotechnology in cancer

## Abstract

UVA1 radiation (340–400 nm), especially longwave UVA1 (> 370 nm), is often ignored when assessing sun protection due to its low sunburning potential, but it generates reactive oxygen species (ROS) and is poorly attenuated by sunscreens. This study aimed to investigate if α-tocopherol phosphate, (α-TP) a promising new antioxidant, could protect against long-wave UVA1 induced cell death and scavenge UVA1 induced ROS in a skin cell model. HaCaT keratinocyte cell viability (24 h) was assessed with Alamar Blue and Neutral Red assays. The metabolism of α-TP into α-T, assessed using mass spectrometry, and the compound's radical scavenging efficacy, assessed by the dichlorodihydrofluorescein (H2DCFDA) ROS detection assay, was monitored in HaCaTs. The mechanism of α-TP ROS scavenging was determined using non-cell based DPPH and ORAC assays. In HaCaT keratinocytes, irradiated with 226 J/cm^2^ UVA1 in low-serum (2%, starved) cell culture medium, pretreatment with 80 µM α-TP significantly enhanced cell survival (88%, Alamar Blue) compared to control, whereas α-T pre-treatment had no effect survival (70%, Alamar Blue). Pre-treatment of cells with 100 μM α-TP or 100 μM α-T before 57 J/cm^2^ UVA1 also significantly reduced ROS generation over 2 h (24.1% and 23.9% respectively) compared to the control and resulted in α-TP bioconversion into α-T. As α-TP displayed weak antioxidant activity in the cell-free assays thus its photoprotection was assigned to its bioconversion to α-T by cellular phosphatases. Through this mechanism α-TP prevented long-wave UVA1 induced cell death and scavenged UVA1 induced ROS in skin cells when added to the starved cell culture medium before UVA1 exposure by bioconversion into α-T.

## Introduction

The impact of terrestrial ultraviolet radiation (UVR) on the human skin, especially shortwave UVB radiation (~ 295–315 nm), is well established. However, the effects of long-wave radiation, i.e., UVA (315–400 nm) are less well understood. Long-wave UVA penetrates deeper into the skin compared to UVB, reaching down into the dermis, and causing photodamage via the generation of reactive oxygen species (ROS) and cyclobutane pyrimidine dimers (CPD)^1–3^. Long-wave UVA1, near the UV–visible light interface (380–400 nm), is emerging as a significant risk to humans because its effects are not visible as ‘sunburn’, which is the main endpoint for sunscreen efficacy^4^. This UVA1 range (380–400 nm) has also previously been shown to induce DNA damage and gene expression of matrix metalloproteinases (MMP1 and MMP9), which are thought to have an important role in photoageing through oxidative reactions^4^ and it has a role in inducing skin hyperpigmentation when the skin is not protected by sunscreens^5^.

Topically applied vitamin E (alpha-tocopherol, α-T) can inhibit UVR-induced DNA damage in human keratinocytes in vitro both pre and post-UVA exposure^6^. However, α-T has several limitations including; (i) poor chemical and photostability^7^, (ii) chromanoxyl radical formation, which leads to deleterious free radical reactions^8,9^, (iii) poor topical formulation solubility due its high hydrophobicity^10^, and (iv) retention in the non-viable outer layer of the epidermis, the *stratum corneum* (SC)^11^. One approach to overcome the limitations of α-T is to use structural derivatives, such as provitamins, that are metabolised by cutaneous phosphatases or esterases to generate α-T. Provitamins can be designed to be chemically stable and less hydrophobic and thus penetrate the SC and pass into the viable skin layers where they can act as antioxidants. α-Tocopherol phosphate (α-TP) is a naturally occurring structural analogue of α-T that is water-soluble, chemically stable and, when formulated as nanoaggregates, can effectively penetrate the SC. Pre-treatment of an ex-vivo cultured mouse skin model with 50 μL of 9 mM α-TP has been shown to protect against UVR (63% UVB and 37% UVA) induced DNA damage and reduce the number of epidermal apoptotic sunburn cells (SBC)^12^. However, to the best of our knowledge, no studies to date have investigated the protection of α-TP against pure long-wave UVA-induced skin damage.

α-TP, when applied topically to the skin, forms nano-sized aggregates that fuse with the SC*,* fluidises it and allows passage into the epidermis where it can act as an antioxidant, or convert into α-T to offer UVR protection^13,14^. The aim of this work was to investigate the photoprotection potential of α-TP against long-wave UVA1-induced cell viability reduction and oxidative stress in an experimental model of HaCaT human keratinocytes.

## Results

### UVA1 source emission and absorption spectra of the test antioxidants

**Figure 1:**
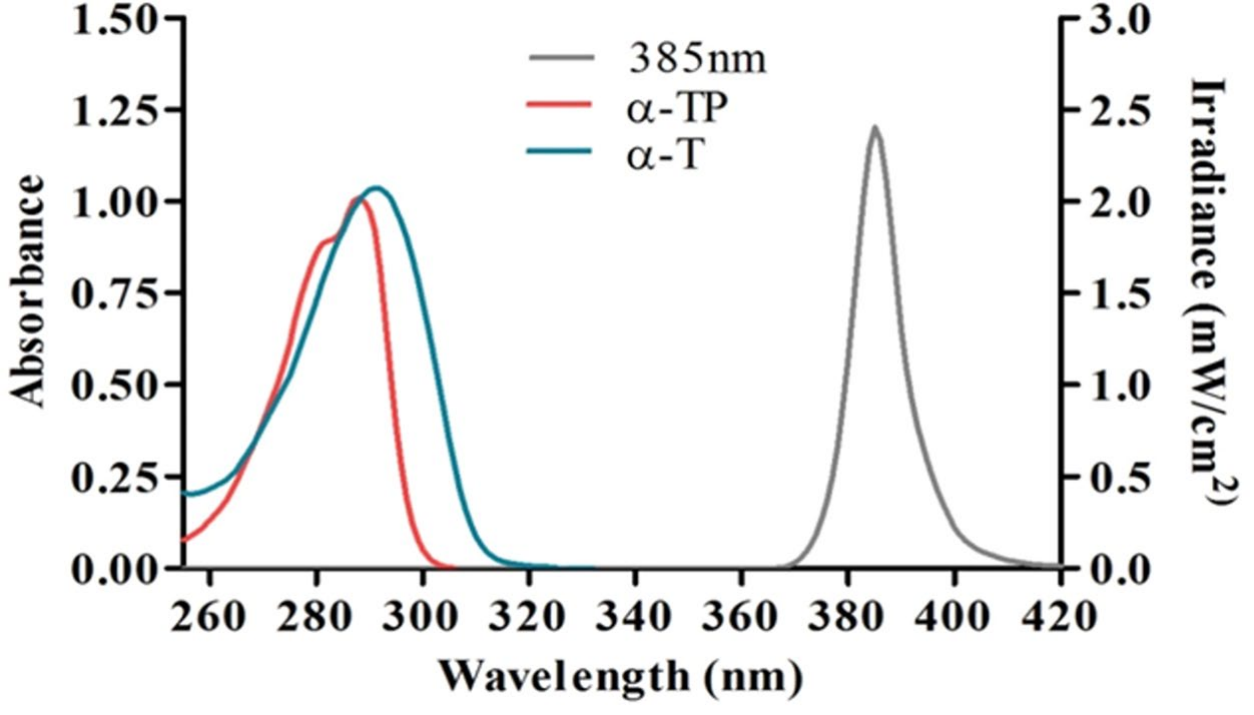
The emission spectrum of the 385 nm UVA1 source (dotted line) and the absorbance spectra test compounds (400 µM) in methanol (solid lines). α-TP and α-T show absorbance peaks in the UVB region at 288 nm and 291 nm, respectively. This excludes any photoprotection by optical filtration.

The UVR emission spectrum (385 nm Loctite UVA) was compared with the absorption spectra of the test compounds (α-TP and α-T) to exclude any possibility of UVR filtering i.e., sunscreening effect. No overlap was seen between the UVR source and either compound (Fig. 1). UVR absorption of α-TP (MW: 554.6 g/mole) and α-T (MW: 431 g/mole) is primarily between 270–320 nm (UVC and UVB) with peaks at 287 nm and 290 nm, respectively.

### Photostability

**Figure 2:**
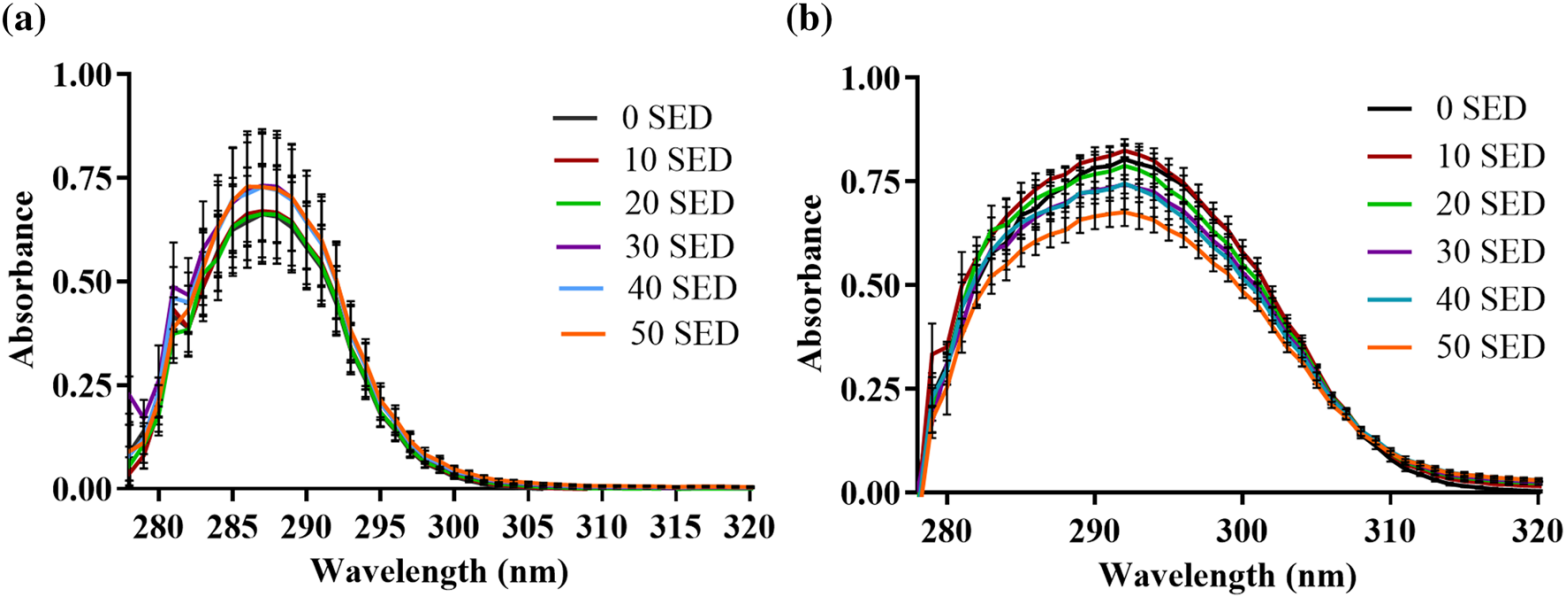
Photostability of α-TP (**a**) and α-T (**b**) when exposed to SSR. Antioxidants (1 mM) were exposed to increasing doses of SSR (10–50 SED) (n = 3). α-TP and α-T displayed very little evidence of photodegradation. Data represent mean ± SD (n = 3).

α-T showed a 14.6% increase in its maximum UVR absorbance at 50 SED compared to 0 SED (*p* = 0.01, paired *t* test, comparing two conditions), but the area under the curve of the absorbance spectra between 290–400 nm showed no significant change (p > 0.05) (Fig. 2 and Table S2). Together these results suggested that although there were some initial signs of chemical degradation, the changes in the absorbance spectra were small and hence α-T showed good photostability under the conditions used in the experiment. The maximum absorbance for α-TP showed no significant change across the data set (p > 0.05), but again there was some slight shifts in the absorbance spectra, which increased the area under the UVA spectral range by almost 50% (Fig. 2 and Table S2). The increase in area under the curve was due to a small red shift and again probably indicated initial signs of chemical degradation, but like α-T there was no drop in UV absorbance and the changes in the absorbance spectra were small and hence α-TP was considered to have good photostability under the conditions used in the experiment.

### Photoprotective compound cell tolerability

**Figure 3:**
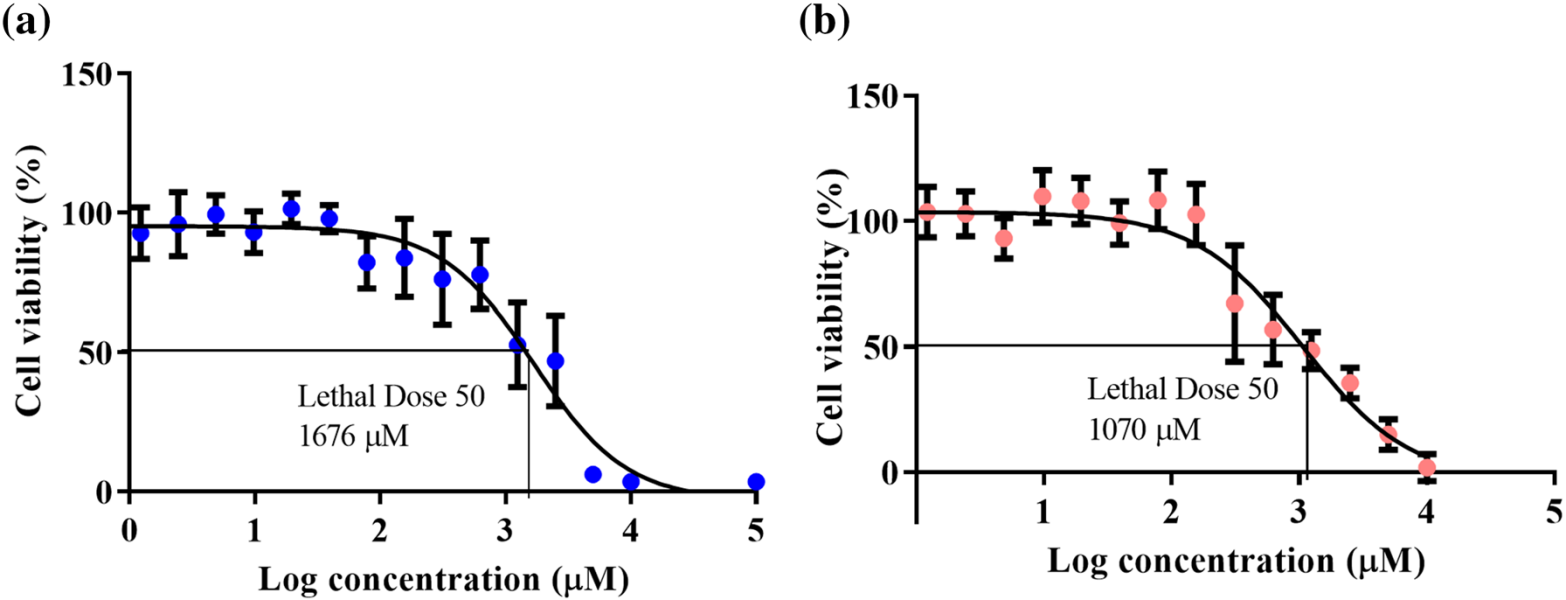
Tolerability of HaCaT keratinocytes to α-TP (0.61–10,000 μM) at 37 °C for 24 h. Lethal dose 50 of α-TP on HaCaT keratinocytes was 1676 μM and 1070 μM using Alamar Blue (**a**) and Neutral Red (**b**) cell viability assays, respectively. Data represent mean ± SD (n = 3).

α-TP was well tolerated in HaCaT keratinocytes and the LD_50_ was comparable using both the Alamar Blue and Neutral Red assays (1676 and 1070 µM, respectively), which suggested that there was no reduction of the resazurin by the test compounds (Fig. 3). α-T was also well tolerated by HaCaT keratinocytes dissolved in 0.5% ethanol in the cell culture medium with no statistical reduction in cell viability after the application of test concentrations 0.61–10,000 μM (Fig. S2 in the supporting information).

### UVA1 dose–response studies

**Figure 4:**
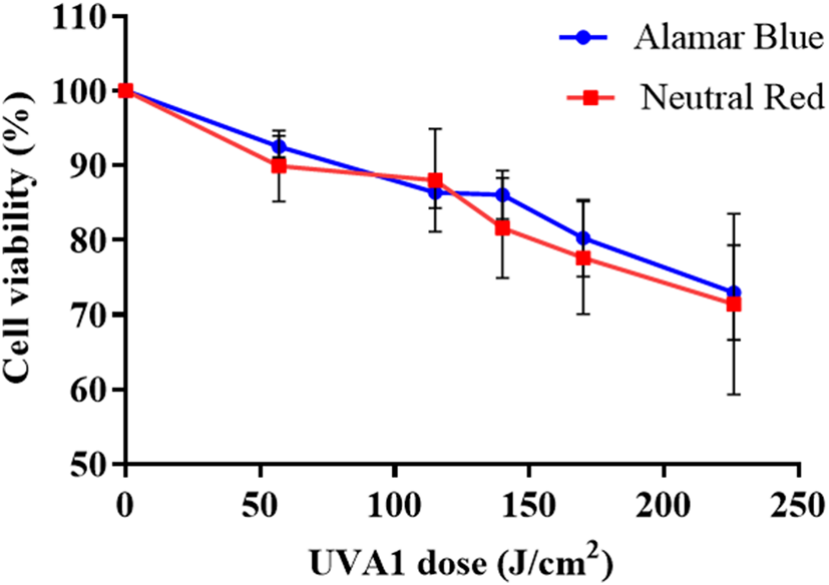
Dose–response cell viability 24 h post irradiation using Alamar Blue and Neutral Red assays. Both assays showed a significant dose dependent decrease in cell viability (Alamar Blue—*p* < 0.0001; Neutral Red *p* < 0.01; n = 3, linear regression analysis) with no significant difference between both assays (*p* > 0.05; n = 3, linear regression analysis). Data represent mean ± SD of three separate experiments.

Alamar Blue and Neutral Red assays again gave comparable results (Fig. 4). For example, after 57 J/cm^2^ the mean cell viability was 92.5% vs. 89.9%; after 115 J/cm^2^ it was 86.4% vs. 88.0%, after 140 J/cm^2^ it was 86.0% vs. 81.6%, after 170 J/cm^2^ was 80.3% vs. 77.6% and after 226 J/cm^2^ it was 71.4% vs. 73.0%, respectively. The UVA1 dose 226 J/cm^2^ was selected to perform the photoprotection studies using cell viability reduction as the endpoint.

### UVA1 cell survival studies

**Figure 5:**
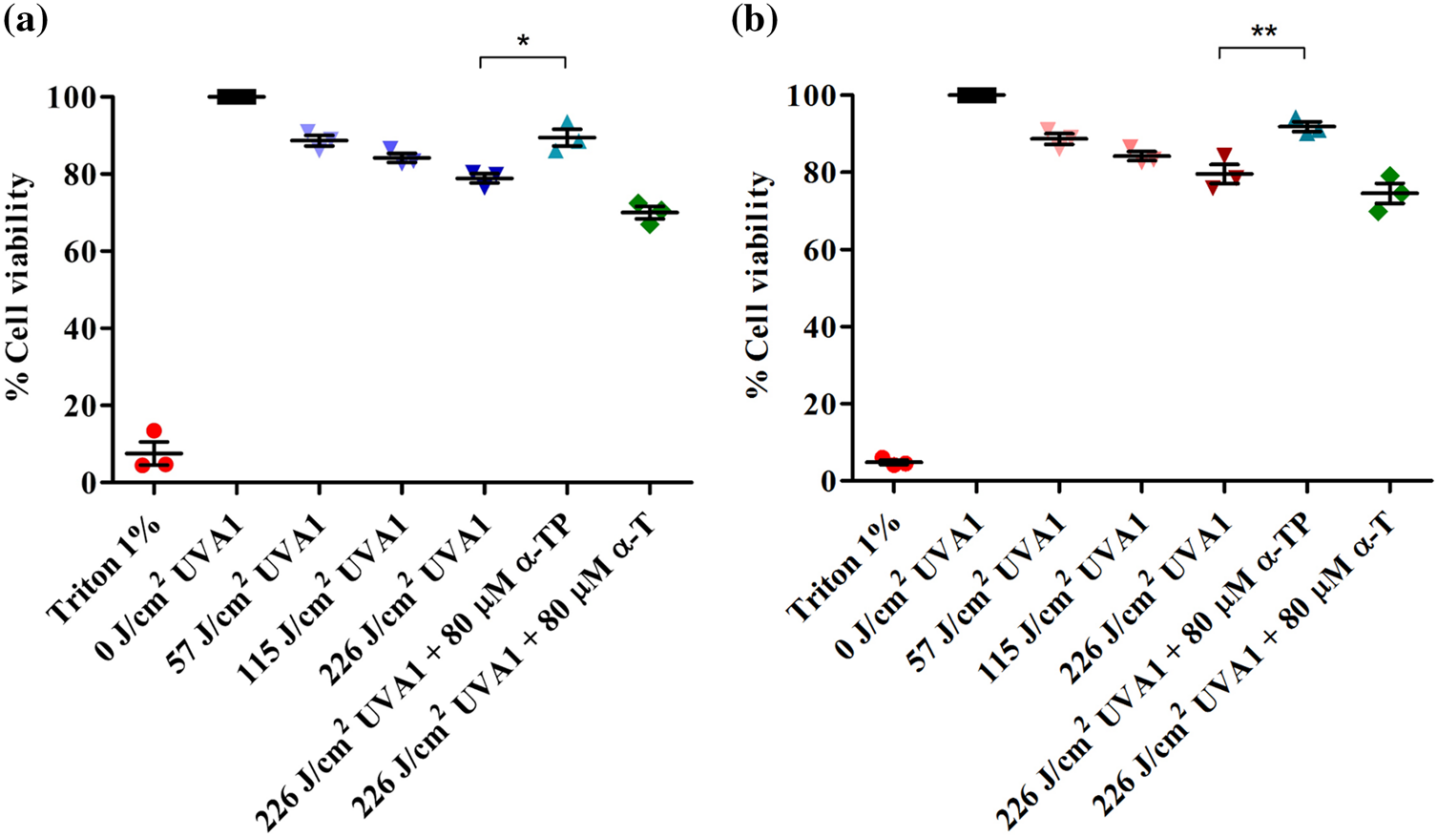
Dose–response experiments for cell viability and effect of pre-UVA1 antioxidant treatment. HaCaT keratinocytes were treated with positive control (Triton 1%), unirradiated, exposed to 57, 115, 226 J/cm^2^ UVA-1 or treated for 24 h with 80 μM α-TP or α-T before UVA1 exposure (226 J/cm^2^). Viability was assessed after 24 h by Alamar Blue (**a**) or the Neutral Red assays (**b**). Note: *p < 0.05. **p < 0.01. Data represent mean ± SD of three individual experiments.

There was a dose dependent reduction in cell viability (57–226 J/cm^2^) after UVA1 irradiation in the absence of any treatment (Fig. 5). When the HaCaT keratinocytes were pre-treated with 80 µM α-T or α-TP in low-serum (2%) medium prior to UVA1 irradiation (226 J/cm^2^), only treatment with α-TP significantly increased the cell survival compared to cells irradiated with the control vehicle (78%) using both Alamar Blue (88%) and Neutral Red assays (93%) (Alamar Blue- p = 0.009, Neutral Red- p = 0.014, one-way ANOVA with Dunnett’s multiple comparisons test) while α-T did not (70–74%) (Fig. 5).

### UVA1 ROS scavenging studies

**Figure 6:**
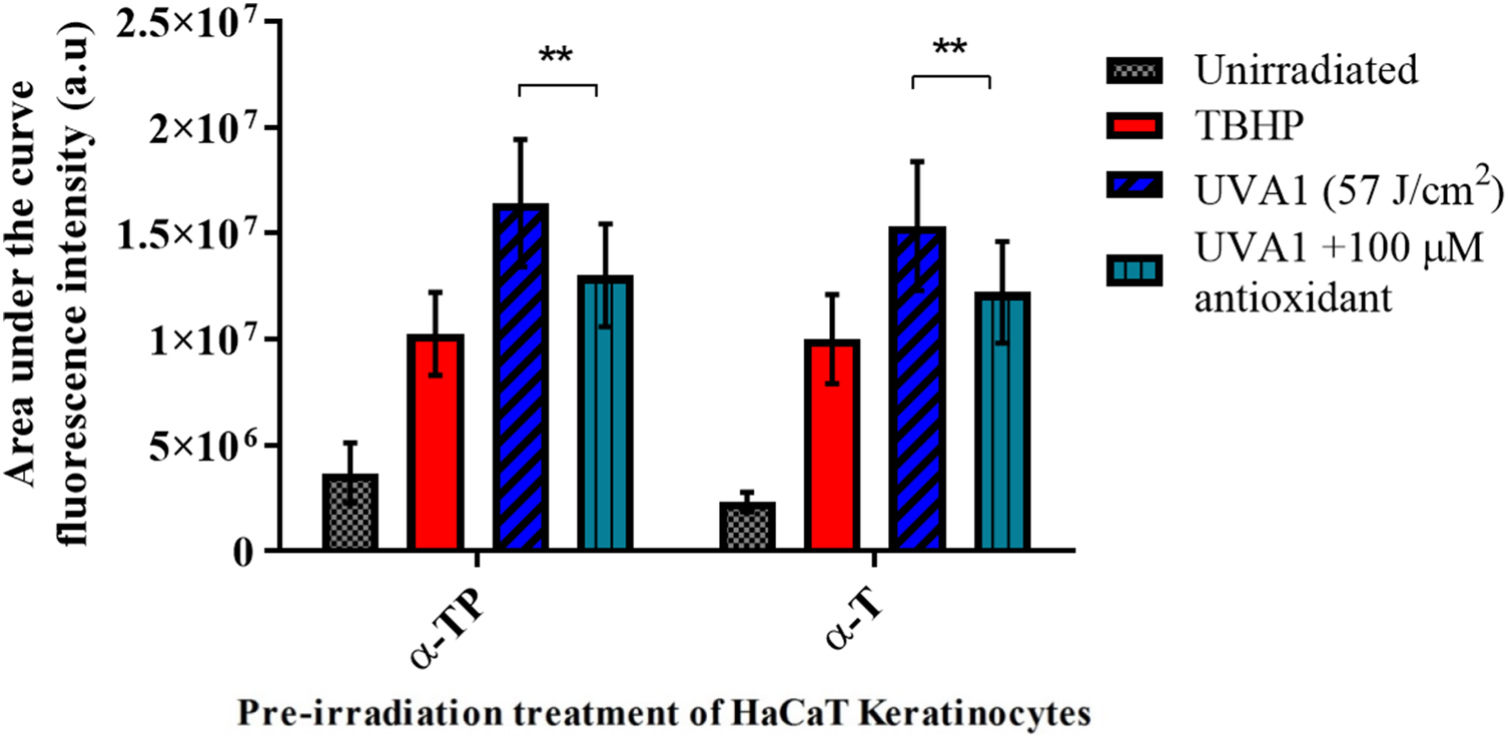
Effect of antioxidants (100 μM) on UVA1-induced ROS in HaCaT keratinocytes. Tertiary butyl hydroperoxide (TBHP—250 μM) was used a positive control. Fluorescence intensity analysis (AUC over 1.8–2 h) taken from Fig. S3 in supporting information (cumulative data). Note: **p < 0.01. Both α-TP and α-T significantly reduced ROS (p = 0.0026, and p = 0.0034 compared to the vehicle control, respectively.

Generation of ROS in HaCaT cells was significantly higher after UVA1 irradiation compared to the unirradiated cells (*p* < 0.0001, paired *t* test, comparing two conditions, for the raw data see Fig. S3 in the supporting information). The positive control, i.e., the chemical generation of ROS using 250 µM TBHP, generated fewer ROS compared to UVA1 (*p* < 0.0001, paired *t* test, comparing two conditions). Pre-treatment of HaCaT with 100 µM α-TP before irradiation with UVA1 caused a significant reduction of ROS generation (ca. 24.1% in ROS generation compared with the vehicle control, *p* = 0.0026, paired *t* test, comparing two conditions). α-T produced a similar reduction in ROS to α-TP (ca. 23.9% reduction in ROS generation, *p* = 0.0034, compared to the vehicle control, paired *t* test, comparing two conditions) (Fig. 6).

### 1,1-Diphenyl-2-picryl-hydrazyl (DPPH) assay

**Figure 7:**
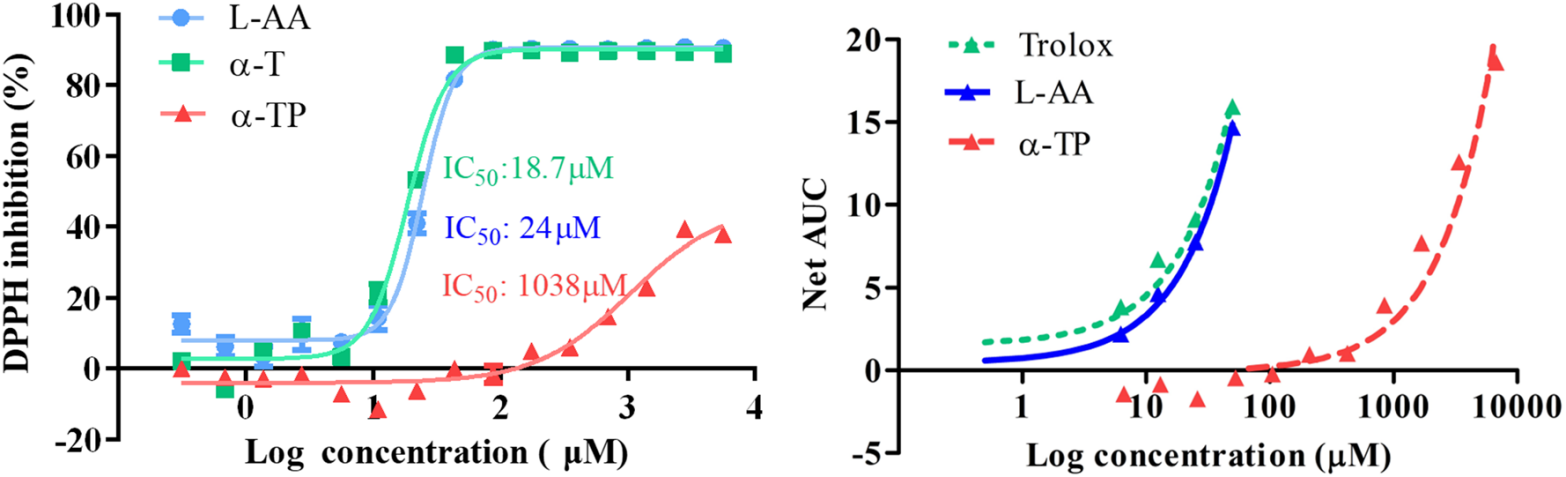
(Left) DPPH radical scavenging ability of α-TP and α-T. l-ascorbic acid (L-AA) was used as a positive control. Aliquots (12.5 μL) of antioxidants at increasing concentrations (0.006–50 mM) were added to wells containing 187.5 μL DPPH (100 μM). IC_50_ values were determined by Graphpad Prism non-linear curve fitting (Gaddum/Schild EC50 fitting). Data represent mean ± SD of three separate experiments. (Right) Ability of α-TP (0–6750 µM) to quench the ROO˙ radical (APPH) using the ORAC assay. Trolox and l-ascorbic acid (0–50 µM) were used as positive controls. Fluorescence degradation over 30 min was assessed with AUC as the readout. Analysis was by linear regression (Trolox: y = 0.2978 x + 1.544, p = 0.0027; l-ascorbic acid: y = 0.2885 x + 0.4473, p = 0.0001; α-TP: y = 0.0042 x + 0.5935, p < 0.0001). 7599 and 107.02 µM of α-TP and l-ascorbic acid respectively were equivalent to 100 µM Trolox. SD were too small to be displayed (n = 3). Ability of α-TP (0–6750 µM) to quench the ROO˙ radical (APPH) using the ORAC assay. Trolox and l-ascorbic acid (0–50 µM) were used as positive controls. Fluorescence degradation over 30 min was assessed with AUC as the readout. Analysis was by linear regression (Trolox: y = 0.2978 x + 1.544, p = 0.0027; l-ascorbic acid: y = 0.2885 x + 0.4473, p = 0.0001; α-TP: y = 0.0042 x + 0.5935, p < 0.0001). 7599 and 107.02 µM of α-TP and l-ascorbic acid respectively were equivalent to 100 µM Trolox. SD were too small to be displayed (n = 3).

The α-TP exhibited poor DPPH activity, with an IC_50_ value of 1038 µM or 575.7 μg/mL (see Fig. S4 in the supporting information). The IC_50_ values of l-ascorbic acid (L-AA) and α-T were 18.7 and 24 µM, respectively using curve fitting (Fig. 7).

### Oxygen radical absorbance capacity (ORAC) assay

α-T was not assessed in the ORAC assay because it is hydrophobic and insoluble in the reaction buffer. Trolox was the reference control because it is a water-soluble vitamin E derivative. The concentrations of α-TP and l-ascorbic acid (L-AA) equivalent to 100 µM Trolox were 7599 and 107.02 µM, respectively (Fig. 7). The order of activity was Trolox > L-AA > α-TP.

### Alpha tocopherol phosphate metabolism

**Figure 8:**
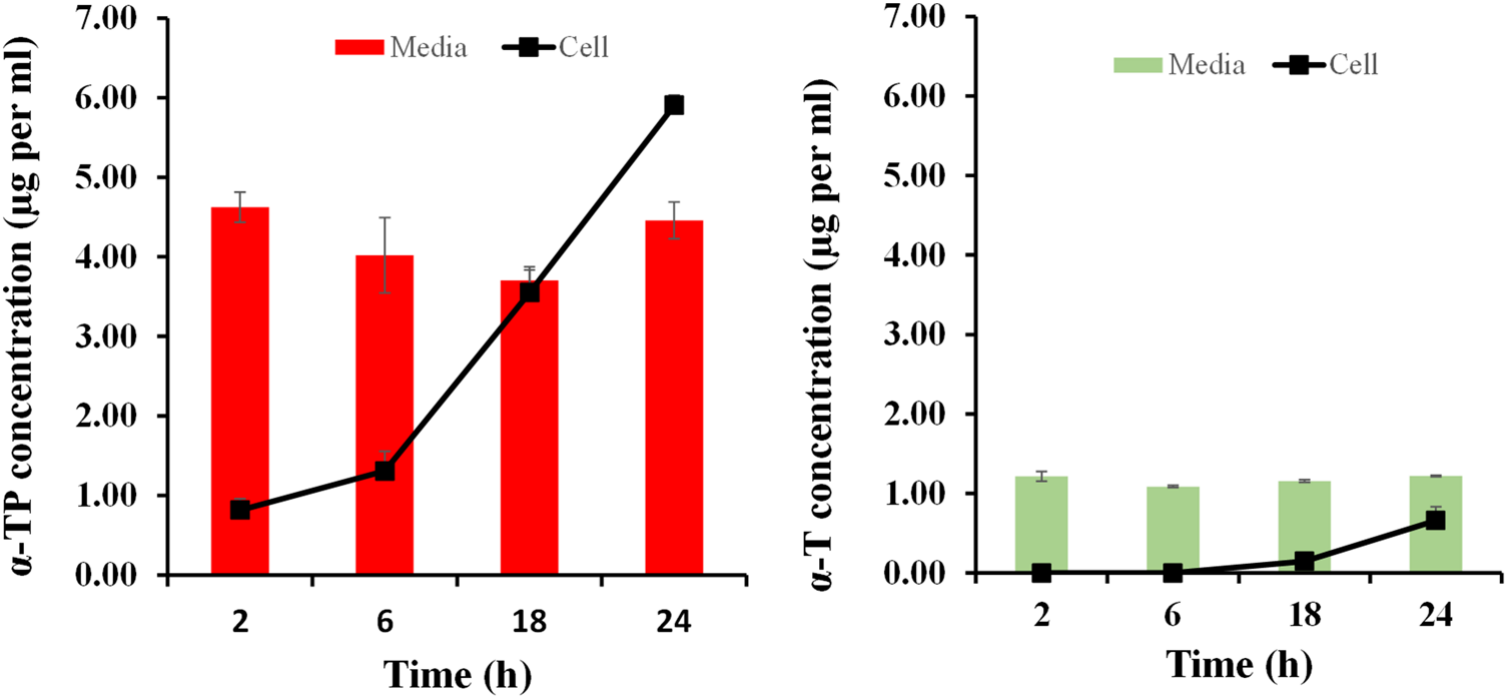
The metabolism of tocopherol phosphate (α-TP) into tocopherol (α-T) in HCAT cells after dosing in cell culture media. (**a**) The concentration of α-TP in the cell culture media and cell lysates over time after the dosing of the cells with 133 µg per mL of α-TP. (**b**) The concentration of α-T in the cell culture media and cell lysates over time after the dosing of the cells with 133 µg per mL of α-TP. Data represents mean ± one standard deviation (n = 3).

The α-TP dissolved in cell culture media applied to the HaCaT cells passed into the cells gradually over the 24 h experiment (Fig. 8). The free α-TP was not depleted in the cell culture media over time demonstrating that a strong concentration gradient is required to drive the compound into the cells. The cell culture media used in the experiments did not contain α-T and hence the appearance of this compound in the cell culture media and the cell lysates demonstrated that α-TP was metabolised into α-T by the HaCATs. The concentrations of α-TP were significantly higher in the cell culture media and the cell lysates compared to α-T across all time points (p < 0.05, ANOVA, Fig. 8). The α-TP concentrations within the cell lysates increased sevenfold over the experiment. In contrast, α-T was not detectable in the cell lysate initially and only increased to 0.7 µg/mL after 24 h, but this only represented cytoplasmic concentration that is readily available as an antioxidant rather than any presence in cell membranes, because an organic extraction process was not used to determine the membrane associated concentrations in this work.

## Discussion

Exposure to UVA1 has been linked to DNA damage that results in formation of CPDs^4^, MMP-1 gene overexpression that may result in photoageing^4^, release of nitric oxide (NO) that may be beneficial for hypertension^22^, and skin opsin-3 upregulation that causes pigmentation^23^. Unlike sunscreens, topical antioxidants have been shown to reduce oxidative damage from ROS induced by UVR without the potential to interfere with vitamin D synthesis. α-T is one of the classical topical antioxidants that has been used to inhibit UVR-induced oxidative damage^24^, it has also been shown to inhibit dark CPD in model systems^6,25^. Such CPD are formed after UVR exposure by a delayed reaction between DNA and reactive compounds, currently believed to be excited carbonyls^25,26^. Dark CPD have been reported in human skin in vivo after 385 nm^4^ and SSR^27^, but their biological significance is unknown.

Numerous limitations of α-T hinder its effectiveness in cosmeceutical products. Adding a phosphate ester to form a provitamin is a strategy that has been shown to overcome these limitations. α-TP has shown important UVA protective properties in the skin, but previous work did not establish if these benefits were due to its antioxidant properties or its ability to act as a sunscreen, as it forms liposomes (with different optical properties) when applied to skin^13^. Furthermore, previous work with α-TP used UVR sources with UVB content that overlapped with the absorption spectrum of α-TP (generated by the compound’s two aromatic rings)^12^. However, to the best of our knowledge no studies to date have investigated the protection of α-TP against spectrally pure UVA-induced skin damage by its antioxidant properties^12^.

The longwave UVA source was selected for a number of reasons. Firstly, given the lack of overlap between the absorption spectra of α-TP and α-T with the UVA1 source it can be concluded that neither compound was capable of absorption/sunscreening effects. Secondly, this is a spectral range that has not been well studied and new LED technology now makes this possible. Thirdly, this is a region known to readily induce oxidative stress with limited induction of other types of damage. Finally, long-wave UVA1 is poorly protected by sunscreens, which focus on preventing the more erythrogenic and shorter wavelengths (< 370 nm). Improved sunscreen protection at shorter wavelengths will result in more exposure in the 385 nm region. As such, there is a need to discover strategies that prevent damage from the longer wavelengths of UVR (370–400 nm).

The UVR absorbance of α-TP slightly increased with increasing SSR dose, most likely due to small changes in molecular aggregation, whereas α-T absorbance did not change, thus both did not showing any significant signs of photodegradation. The SSR dose in this study was 51 J/cm^2^, which corresponds to 3.4 h of UK summer sun, previous reports that showed α-T photodegradation used a much higher dose of 198 kJ/m^2^ (19.8 J/cm^2^) using SSR (290–500 nm) and resulted in a subsequent loss of the compound’s antioxidant activity^28^.

α-TP reduced HaCaT keratinocyte viability more than α-T (LD_50_ was 1.1–1.8 mM vs > 4.70 mM, respectively) suggesting the phosphate group enhanced the molecule’s ability to enter the cells and cause cytotoxicity. This has also been shown in oral mucosal cells in a study that hypothesised that toxicity was caused by disruption of intracellular enzymes^29^. Previous studies in other cells lines support selective cell toxicity by α-TP, but α-T is generally well tolerated^30^. However, the cytotoxicity of TP strongly depends on its isomeric form with the γ-form showing better growth inhibitory activity than δ-form (IC_50_ was 30 vs. 55 µM, respectively) in human colorectal cancer cells (HCT116 and HT29 cells). In the same study, the α-form was the least inhibitory compared to other isomers (IC_50_ > 100 µM)^31^.

Interestingly, pretreatment of human HaCaT keratinocytes with α-TP in starved low serum media (2% FBS) for 24 h exhibited superior protection against long-wave UVA1 (385 nm) induced cell death compared to α-T. A similar effect has been reported by Nakayama and colleagues who found that incubation of skin cells with 2% FBS significantly depleted endogenous antioxidant α-T by 50% after 24 h incubation. They also showed that treatment with 0.5% α-TP dissolved in 2% DMEM not only inhibited depletion of endogenous α-T, but also increased its level by two-fold compared to untreated cells. In the same study, UVB irradiation was found to significantly reduce endogenous α-T in cultured skin by 50% compared to unirradiated skin. This reduction was inhibited by pre-UVB treatment with α-TP for 3 h which caused a two-fold rise in α-T compared to irradiated cells treated with vehicle control^12^. This may be due to the release of α-T from the provitamin E within keratinocytes by their phosphatase enzymes, which prolongs the protection effect even after 24 h. Another possible reason is that the incorporation of α-TP is essential to prevent the propagation of radical species^32^.

It is well known that ROS are significantly increased in cells during and following UVA irradiation^33,34^. As UVA-related biological effects are largely mediated by ROS, their elimination is essential for protection against UVA damage. In this work, pre-irradiation treatment with either α-TP or α-T result in reduction in UVA1-induced ROS generation in HaCaTs to a similar extent (by ~ 24.1% and 23.9%, respectively) compared to the irradiated vehicle control treated cells. Wu et al. investigated photoprotection of HaCaT keratinocytes using UVA1 (365 nm) and monitored ROS generation. They reported that 2.9 IU/mL of α-T in serum free medium significantly reduced (80%) ROS generation compared to irradiated vehicle treated cells (742.5 vs. 3952.2 fluorescence intensity/protein, 1/mg protein, p < 0.05)^24^. The difference in the degree of ROS suppression with our findings using the dichloro-dihydro-fluorescein (DCFDA) assay, may be due to the differences in UVA1 source (365 nm vs. 385 nm) and UVA1 dose (8 J/cm^2^ vs. 57 J/cm^2^), the concentration of tested compound (2 mg/mL = 4.6 mM vs. 100 µM), the concentration of H_2_DCFDA (5 µM vs. 20 µM), and the level of supplements in growth medium (serum-free vs. low-serum medium).

Studies were performed with DPPH and ORAC assays without cells to determine if α-TP afforded innate antioxidant activity or was the outcome of its potential bioconversion to α-T. As free radical scavenging by antioxidants can proceed through single electron transfer (SET) or hydrogen atom transfer (HAT), two assays (DPPH and ORAC respectively) were used to identify the mechanisms^35^. α-TP exhibited poor DPPH activity compared to ascorbic acid and α-T. The α-T IC_50_ of 19 µM was lower than previously reported at 40.6 μM, but within normal inter-laboratory variability^36^. The ascorbic acid IC_50_ of 24 µM was slightly lower than previously reported, i.e., 34 µM^37^, 54.6 µM^38^, 49.5 µM^36^, but confirmed that assay worked well. This poor activity suggested that α-TP had very little antioxidant capacity per se via SET whereas α-T and ascorbic acid were potent antioxidants via this mechanism. α-TP and water-soluble ascorbic acid were compared to Trolox in the ORAC assay. Trolox activity was almost equivalent to ascorbic acid, but was 75-fold more effective than α-TP. Our findings agree well with those of Rezk et al. (2004) using the Trolox equivalent antioxidant capacity (TEAC) assay, in which they determined the TEAC for Trolox and α-TP (1.0, and 0.01, respectively), i.e., Trolox was 100-fold more potent than α-TP^39^. Weak α-TP activity in two different assays indicated that bioconversion to α-T was probably responsible for the UVR protective in a biological model.

The metabolism studies were designed to investigate the availability of α-TP and α-T in the cell culture media and cell cytoplasm over time after the dosing of the cells with α-TP. No extraction of the agents from the cell membrane was performed as it was expected that the metabolism studies would explain the α-TP intracellular ROS savaging observed after UVA1 exposure despite its weak in vitro activity. The µg/mL detection of α-T in both the cell culture media and the cell lysate after α-TP, whilst not being present in the control experiments that only applied cell culture media, demonstrated that the cells did metabolise the α-TP into α-T. Previous work in THP-1 cells has shown that α-TP does metabolise into α-T and that the concentration of α-T found in the cells was around 3% of the initial α-TP added^40^. In the HaCaT cells only 0.4% of the applied α-TP converted into α-T and passed into the cell cytoplasm. In the current study LC–MS was employed to specifically detect cytoplasmic α-T with no lipid extraction method being applied, whereas in the previous work radiochemical detection was employed with a lipid extraction method. It is reasonable to suggest that lipid extraction coupled with the radiochemical method would account for the membrane associated α-T as well was that in the cytoplasm, and this provides an explanation for the differences in the reported values. The metabolism studies also suggested that the α-TP penetrated the cells more quickly and to a greater extent compared to α-T. This is in accordance with our own previously published work, which demonstrated that α-TP penetrates the skin more readily than α-T, because the latter is trapped within the lipid regions of the cell membranes^15^. This work was able to demonstrate this effect as it did not mimic the published methods to study tocopherol metabolism as they also extracted the compounds from the cell membranes, which can overestimate the α-T available to quench ROS within the cell.

A limitation of this work is that it was performed in immortalised HaCat keratinocytes rather than primary keratinocyte cell lines or more complex skin models. It can be problematic to study oxidative stress in complex systems due the tissue isolation or growth processes inducing oxidative stress. The use of HaCaTs has been established as valid in photobiological research^41–44^, and the aim of this study was to determine the relative antioxidant effects of the test compounds at the cellular level given our previous work had demonstrated that α-TP reaches the epidermis after topical administration. Also, we have found in our previous studies a good correlation between the HaCaT model and human in vivo studies^45^.

In conclusion, α-TP displayed weak antioxidant activity when assessed by chemical assays. In HaCaT keratinocytes it was well tolerated, and had superior photoprotection properties compared to α-T. Pre-irradiation treatment of HaCaT keratinocytes with α-TP resulted in a similar reduction in ROS generation as α-T, but α-TP penetrates more effectively into the cells and the skin compared to α-T. The biological activity of α-TP was found to be a consequence of its bioconversion to α-T by endogenous cellular phosphatases, which increase the intercellular level of α-T even after UVA1 exposure ends. That α-TP proved to be a photostable provitamin E form that has greater protection against cell viability reduction and comparable reduction in level of ROS generated by UVA1 exposure compared to the parent molecule and thus it is an excellent candidate to include in sun-protection products to reduce the impact of UVA1-induced skin damage.

## Materials and methods

### Materials

α-TP ( (+/-)α tocopherol phosphate, purity: 92.7%, cosmetic grade) was supplied by Shawa Denko (Tokyo, Japan). Natural (+) α-T (type VI, purity: 70%, 695 mg d-α-tocopherol per g, 1036 IU/g), Neutral Red, and Corning cell culture flasks were sourced from Sigma-Aldrich (Dorset, UK). Dulbecco modified Eagle’s medium (DMEM, high glucose, no pyruvate, no glutamine), foetal bovine serum (FBS), l-glutamine, penicillin–streptomycin, phosphate-buffered saline (PBS) pH 7.4, TrypLE™ Express (1 ×, phenol red-free) were sourced from Thermofisher Scientific (Paisley, UK). Alamar Blue was purchased from Fisher Scientific (Leicestershire, UK). Triton-X was supplied from Promega (Southampton, UK). Plastic bottom black walled (to prevent UVR scatter) 96 well plates were purchased from PerkinElmer (Beaconsfield, UK). The 2′,7 dichloro-dihydro fluorescein diacetate (H_2_DCFDA) cellular ROS detection assay kit was sourced from Abcam (Cambridge, UK). The oxygen radical absorbance capacity (ORAC) antioxidant assay kit was supplied by Zenbio (North Carolina, USA). The 1,1-Diphenyl-2-picryl-hydrazyl (DPPH) assay was sourced from Alfa Aesar (Lancashire, UK). In all the studies, correction of natural α-T to account for its 70% purity was not performed because the biological activity of natural 70% pure (+) α-T (1036 IU/g) was equivalent to the pure synthetic α-T (1100 IU/g) and this allowed easy comparison. It is accepted that some impurities are present in the natural α-T and that these may have photoprotection activity, but this was thought to be unlikely.

### Radiation sources

The UVA1 source was a Loctite LED flood system with a peak (λmax) at 385 nm (Loctite, Henkel Ltd, Hemel Hempstead, UK). The array has an irradiation surface of 97 mm × 96 mm consisting of 144 LEDs. A 300 W-16S xenon arc solar UVR simulator (Solar Light, Glenside, PA, USA) with a full solar spectrum UVR setting, complying with ISO Standard 24444 and COLIPA (now Cosmetics Europe) 2006, was the source of solar-simulated radiation (SSR). The spectral irradiances of the sources were measured as previously described^4,15^ and are shown in Fig. S1 and outputs are described in Table S1 in the supporting information. The 385 nm spectrum was measured at a distance of 40 cm and the SSR spectrum was measured at a distance of 0 cm. Irradiation distances were based on the output of the source being measured and were selected to be within the dynamic range of the spectroradiometer. Irradiances were routinely measured with hand-held radiometers. This was a Loctite UVA/Vis radiometer (Loctite, Henkel Ltd, UK) for the 385 nm source. A typical irradiance of 88.5 mW/cm^2^ with irradiation time of 11 min gave a dose of 56.5 J/cm^2^. The solar simulator spectrum was measured using a Solar Light PMA 2100 radiometer (Solar Light, Glenside, Pennsylvania). The solar simulator was calibrated using spectroradiometric readings such that an irradiance of 1100 μW/cm^2^ for 18 s gave 2–3 standard erythema dose (SED).

### Absorption spectra of photoprotective compounds

α-TP and α-T were prepared at a concentration of 400 µM in methanol. Their UVR absorbance spectra were determined with a Perkin Elmer Lambda 2 UV/VIS Spectrometer (Perkin Elmer & Co GmbH, Oberlingen, Germany) between wavelengths 250–420 nm, to assess for possible sunscreening effects.

### Photostability

SSR was used to test photodegradation of 1 mM (0.05% w/v) α-TP dissolved in Tris buffer pH 7.4 and an equivalent molar concentration of α-T dissolved in ethanol using increasing doses of SSR (10–50 SED or 17.8–88.9 J/cm^2^). Protected samples were used as dark controls (wrapped with aluminium foil). Absorbance of the dark controls A_0_ and samples (A_T_) was measured between 280–400 nm (n = 3) after each exposure using a FLUOstar-Omega microplate reader (BMG LABTECH Offenburg, Germany). Percent degradation was calculated from the ratio of the change in absorbances (A_T_ − A_0_) after exposure and the absorbance of dark control (A_0_) at 288 nm for α-TP and 291 nm for α-T, after normalising by subtracting the blank absorbance.

### Cell culture

The immortalised human HaCaT keratinocyte cell line was purchased from the American Type Culture Collection (ATCC, Manassas, VA, USA). This cell line has two p53 spontaneously transformed point mutations. HaCaT cells were cultured in DMEM supplemented with 10% FBS, 5% penicillin–streptomycin, and 5% glutamine, maintained in a humidified incubator at 37 °C with 95% air and 5% CO_2_. Cells were cultured to around 80% confluence in 75 cm^2^ plastic flasks (Corning, New York, USA). Cells were plated into plastic bottom black wall 96 well plate (Perkin Elmer) and left to reach a confluence of 70–80% before being used for experiments.

### Cell tolerance of photoprotective compounds

The cytotoxic potential of α-TP and α-T was evaluated using the Alamar Blue^16^ (Fisher Scientific, Loughborough, UK) and Neutral Red^17,18^ (Sigma-Aldrich, Dorset, UK) viability assays in HaCaT keratinocytes. Alamar Blue is a metabolic function (redox) indicator, which measures viable cells’ ability to reduce resazurin, to fluorescent resorufin. The Neutral Red assay assesses the ability of viable cells to incorporate the cationic dye into their lysosomes. Two assays with different mechanisms of action were employed to strengthen conclusions. The cells were treated for 24 h with α-TP (0.0001–10 mM) in prewarmed cell culture media at 37 °C (cell culture media was used as a control) or α-T (0.0001–5 mM) in 0.5% ethanol mixed with prewarmed full media at 37 °C (0.5% ethanol in cell culture media was used as a control). The test compounds were aspirated from each well and the culture media containing 1:10 Alamar Blue or 1:100 Neutral Red solution was added to each well for 1.5 h or 3 h, respectively. The Alamar Blue fluorescence intensity was measured directly at excitation/emission = 570/585 nm using an Infinite 200 PRO spectrofluorometer (Tecan Group Ltd, Männedorf, Switzerland). For Neutral Red, cells were washed three times in PBS to remove excess stain and then de-stain solution [50% v/v ethanol, 49% ddH_2_O, 1% glacial acetic acid (GAA)] was added. Neutral Red optical density was measured at 540 nm. Cell viability for α-T or α-TP treated cells was calculated as a percentage of the control value. Each condition was tested in triplicate from three different passage numbers ranging from 16–20 passages.

### UVA1 dose–response studies

HaCaT viability was determined over a range of 385 nm doses (56.5–225.9 J/cm^2^). This was determined after irradiation using Alamar Blue and Neutral Red assays with a FLUOstar-Omega microplate reader (BMG LABTECH, Offenburg, Germany). The viability was calculated as a percentage of the unirradiated value.

### UVA1 cell survival studies

Cell starved low-serum (2%) media was used, as reported by Nakayama, to reduce the level of endogenous α-T^12^. HaCaT cells, were added to black-walled 96-well plates (10,000 cells per well) and left overnight to adhere. They were washed twice with warmed PBS (100 μL/well) and treated with 80 µM of α-TP (1.5% tris buffer in medium was used as a control) or α-T (0.5% ethanol in medium was used as a control), and then incubated at 37 °C with 5% CO_2_ for 24 h. After aspiration of treatment solutions, the cells were washed twice with warmed PBS (100 μL/well), then fresh PBS (100 μL/well) was added to each well and the cells were irradiated with UVA1 without the plate lid. Each well was exposed individually for up to 44 min to obtain a maximum UVA1 dose of 226 J/cm^2^. Cells were kept on a cooling platform to keep them at around 37 °C. Unirradiated controls (covered with foil) were kept in the same conditions as the longest exposure time to ensure any differences observed were due to the UVA1 exposure rather than any confounding factors. The cells were washed, and the cell viability was assessed using both the Alamar Blue and Neutral Red assays 24 h post-irradiation.

### UVA1 ROS scavenging studies

HaCaT keratinocytes were seeded at a concentration of 25,000 cells per well in a black-walled, clear bottom 96-well plate and left for 24 h to adhere. They were washed in PBS and pretreated with 100 µL of a 100 µM α-TP or α-T for 24 h and then immediately washed with 1 × PBS followed by incubation with 100 µL of a 20 µM DCFDA solution in the dark for 45 min. The cells were then washed with PBS followed by UVA1 irradiation [57 J/cm^2^ (10 min exposure)]. Tertiary butyl hydroperoxide (TBHP) was added into the assigned positive control wells at concentration 250 µM. Fluorescence intensity from cells of each well was measured using a FLUOstar-Omega microplate reader (BMG LABTECH, Offenburg, Germany) with excitation/emission = 485/520 nm. The mean (n = 3) area under the curve (AUC) fluorescence intensity over 2 h was calculated for each condition. The mean difference in AUC fluorescence intensity between treatments, and the unirradiated vehicle control were calculated.

### 1,1-Diphenyl-2-picryl-hydrazyl (DPPH) assay

DPPH is hydrophobic dye that participates in hydrogen atom transfer (HAT) reactions, but strong hydrogen-bonding solvents such as methanol interfere with release of hydrogen atoms and thus enhance single electron transfer (SET) over HAT^19–21^. A series of DPPH (6.25–0.0008 mM) solutions was prepared in the solvent in which the test compound was dissolved, (ascorbic acid was dissolved in DMSO, α-T was dissolved in methanol, and α-TP was dissolved in water). Aliquots of these samples were transferred to a 96-well plate, protected from light, placed on a shaker at room temperature for 30 min and their absorbances were measured at 517 nm using an Infinite 200 PRO spectrofluorometer (Tecan Group Ltd, Männedorf, Switzerland). Each condition was tested in triplicate. The average percentage inhibition of DPPH was calculated. Linear regression analysis (inhibition vs. concentration) was carried out to calculate the effective concentration for 50% inhibition (IC_50_) for each compound.

### Oxygen radical absorbance capacity (ORAC) assay

The ORAC assay tests a compound’s ability to inhibit peroxyl radicals (ROO·) from oxidising fluorescein. This assay was carried out with the ORAC Antioxidant Assay Kit (Zenbio, Research Triangle Park, North Carolina, USA) according to the manufacturer’s instructions. Trolox standards were prepared in the assay buffer (0–100 µM) along with serial dilutions of the test compounds. The fluorescein working solution (150 µL) was added to the wells of a 96 well plate, with 25 µL of each of the standards or test compound in duplicate, and the plate was incubated at 37 °C for at least 15 min. A 2,2′-azobis-2-methyl propanimidamine dihydrochloride (APPH) working solution was then added to each well (25 µL) to start the reaction. Fluorescence was measured in a preheated incubation chamber (37 °C) using an Infinite 200 PRO spectrofluorometer (Tecan Group Ltd, Männedorf, Switzerland) with excitation/emission = 485/530 nm taken immediately and then every minute for 30 min. Standard curves were generated for each compound and the area under the curve (AUC) calculated. Each compound tested was then expressed as a Trolox equivalent concentration.

### Alpha tocopherol phosphate metabolism studies

HaCaT keratinocytes were plated (9 × 10^5^ cells per well), in triplicate, into a 6 well plate using 1 mL of media. α-TP 6 mM stock solution was made in 0.1 M tris buffer, diluted in DMEM, and (1 mL, 500 μM) was applied to groups of triplicate cells that were incubated either for 2 h, 6 h, 18 h or 24 h (final conc. 250 μM, eq 133 µg per mL). Samples were taken from the cell culture media and the cells were harvested. Cells were then lysed through three freeze–thaw cycles and mechanical syringe homogenisation to release their contents. Lysates were centrifuged to remove cell debris, transferred to a fresh Eppendorf tube and snap frozen at − 80 °C. When ready for analysis, the lysates and cell culture media samples were thawed and the internal standard tocopherol acetate (50 µg/mL), dissolved in acetonitrile, was added. The acetonitrile precipitated the protein in the samples, and they were centrifuged. The supernatants were passed through a solid phase extraction plate (Oasis HLB, Waters, UK) preconditioned using phosphoric acid and methanol. The analytes adsorbed to the solid phase extraction plate were washed with 20% methanol 80% water and eluted into a 96 well plate using 200 µL of isopropyl alcohol. A series of calibration standards were subjected to the same processes as the cell samples to quantify αT and αTP. The LC–MS analysis used a Waters Aquity ultra high-performance liquid chromatography and Xevo TQ MS quadrapole mass spectrometer (Waters, UK). A Waters Aquity BEH C18 1.7 µm, 2.1 mm × 50 mm column (Waters, UK) was employed for the analysis with a mobile phase comprising 90% isopropyl alcohol: 10% water containing 0.1% formic acid. The mass spectrometer settings were optimised for each compounds, but each used a source temperature of 150 °C, a desolvation temperature of 500 °C, a desolvation gas flow of 600 L/h and a capillary flow of 3.9 kV. α-TP was detected using negative ESI using MRM transition of 509.22 > 78.88 with a cone voltage 34 and collision energy of 24. α-Twas detected using positive ESI using the MRM transition 431.22 > 164.99 with a cone voltage 26 and collision energy of 22 and alpha tocopherol acetate using the MRM 490.40 > 207.0 with a cone voltage 26 and collision energy of 50.

### Statistical analysis

All data are expressed as the mean ± standard deviation (SD) where n = 3 unless stated otherwise. The homogeneity of variance (Levene’s test) and the normality (Shapiro–Wilk test) of all sample group data were assessed prior to statistical analysis that was performed using the Statistical Package of Social Sciences program, SPSS version 17 (IBM Corp., USA) with a significance level of 0.05. Comparisons were performed using the Student’s *t* test, paired *t* test comparing two conditions, ANOVA with multiple comparisons tests (Tukey’s, or Dunnett’s test), linear regression, non-linear regression. Data are presented using Prism software (GraphPad Prism, version 5.02, December 2008).

## Supplementary Information

Supplementary Information.

## Data Availability

The datasets generated during and/or analysed during the current study are available from the corresponding author on reasonable request.
