# Supplementary material for: The photoprotective properties of α-tocopherol phosphate against long-wave UVA1 (385 nm) radiation in keratinocytes in vitro
Source: Sci Rep. 2021 Nov 17;11:22400. doi: 10.1038/s41598-021-01299-y (PMC8599454; doi:10.1038/s41598-021-01299-y)
Supplement: Supplementary file 1 — Supplementary Information. [file 41598_2021_1299_MOESM1_ESM.docx]

**The photoprotective properties of α-tocopherol phosphate against long-wave UVA1 (385 nm) radiation in keratinocytes *in vitro***

M M Saleh ^a^, K P Lawrence ^b^, S A Jones ^*c^ and A R Young ^b^

^a^ Department of Pharmaceutics and Pharmaceutical Technology, School of Pharmacy, The University of Jordan, Amman 11942, Jordan

^b^ King’s College London, St John’s Institute of Dermatology, Guy’s Hospital, London, SE1 9RT, UK

^c^ Institute of Pharmaceutical Science, Faculty of Health Sciences & Medicine, Franklin-Wilkins Building, King's College London, 150 Stamford Street, London SE1 9NH, UK

*Corresponding author: Dr. S. A. Jones. King’s College London. School of Cancer and Pharmaceutical Sciences, Faculty of Life Sciences & Medicine, Franklin-Wilkins Building, 150 Stamford Street, London SE1 9NH. Tel: +44 (0)207 848 4843. Fax: +44 (0)207 848 4800. Email: stuart.jones@kcl.ac.uk

# Supporting Information


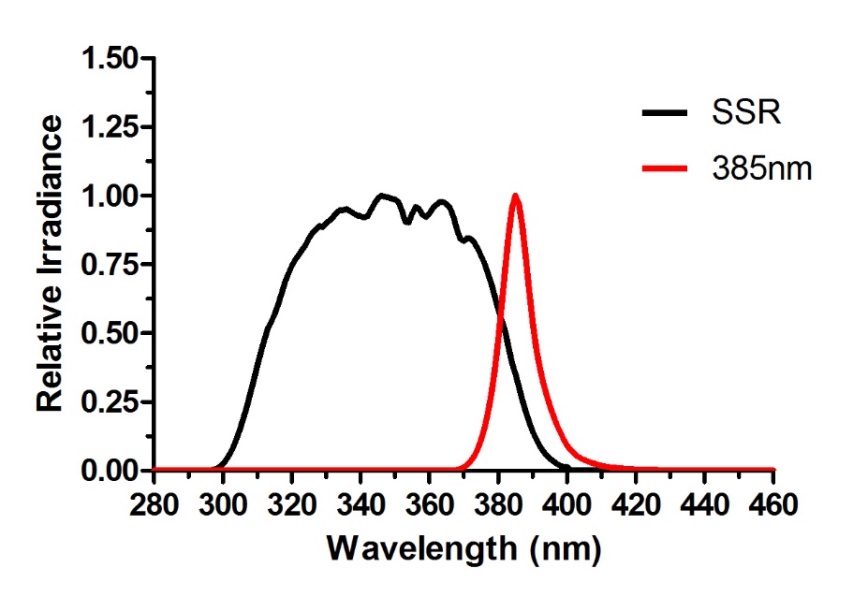


**Fig.S1: The spectral outputs of the radiation sources.** The spectral output of the sources used in all the studies as measured with a Bentham spectroradiometer (details below in dosimetry section) from 280-460 nm.

**Table S1: Spectral waveband analyses of the radiation sources.** The spectral breakdown of each of the sources used in all the studies as measured.

| Source | Region | Wavelength (nm) | % of total irradiance |
| --- | --- | --- | --- |
| SSR | UVC | 250-280 | 0 |
|  | UVB | 280-320 | 12 |
|  | UVA | 320-400 | 88 |
|  | Visible | 400-500 | 0 |
|  | Total | 280-500 | 100 |
|  | | | |
| 385 nm | UVC | 250-280 | 0 |
|  | UVB | 280-320 | 0 |
|  | UVA | 320-400 | 94.82 |
|  | Visible | 400-500 | 5.18 |
|  | Total | 280-500 | 100 |

**Table S2: The doses of SSR used and percentage reduction in absorbance for α-TP and α-T photostability study.** The equivalent doses (J/cm^2^) for each dose (SED) used to test the photostability of 0.5 mg/mL (1 mM) α-TP or 0.43 mg/mL (1 mM) of α-T. The absorbance at the peak and the area under the curve (AUC) between 290 – 400 nm stated. Number are mean ± 1 SD (n=3).

| Dose (SED) | Exposure time | Dose (J/cm^2^) | α-T Abs. at 288 nm | α-T AUC 290 nm – 400 nm | α-TP Abs. at 291 nm | α-TP AUC 290 nm – 400 nm |
| --- | --- | --- | --- | --- | --- | --- |
| 0 | 0 s | 0 | 0.92 ± 0.02 | 10.70 ± 1.20 | 0.78 ± 0.08 | 3.07 ± 0.04 |
| 10 | 70 s | 17.8 | 0.92 ± 0.02 | 11.44 ± 0.53 | 0.81 ± 0.04 | 3.33 ± 0.20 |
| 20 | 140 s | 35.6 | 0.93 ± 0.03 | 11.30 ± 1.10 | 0.77 ± 0.82 | 3.50 ± 0.24 |
| 30 | 210 s | 53.3 | 0.93 ± 0.02 | 11.10 ± 0.38 | 0.73 ± 0.04 | 3.66 ± 0.17 |
| 40 | 280 s | 71.1 | 1.01 ± 0.03 | 10.96 ± 1.33 | 0.73 ± 0.11 | 4.54 ± 0.49 |
| 50 | 350 s | 88.9 | 1.02 ± 0.05 | 10.34 ± 0.82 | 0.67 ± 0.06 | 4.57 ± 0.40 |


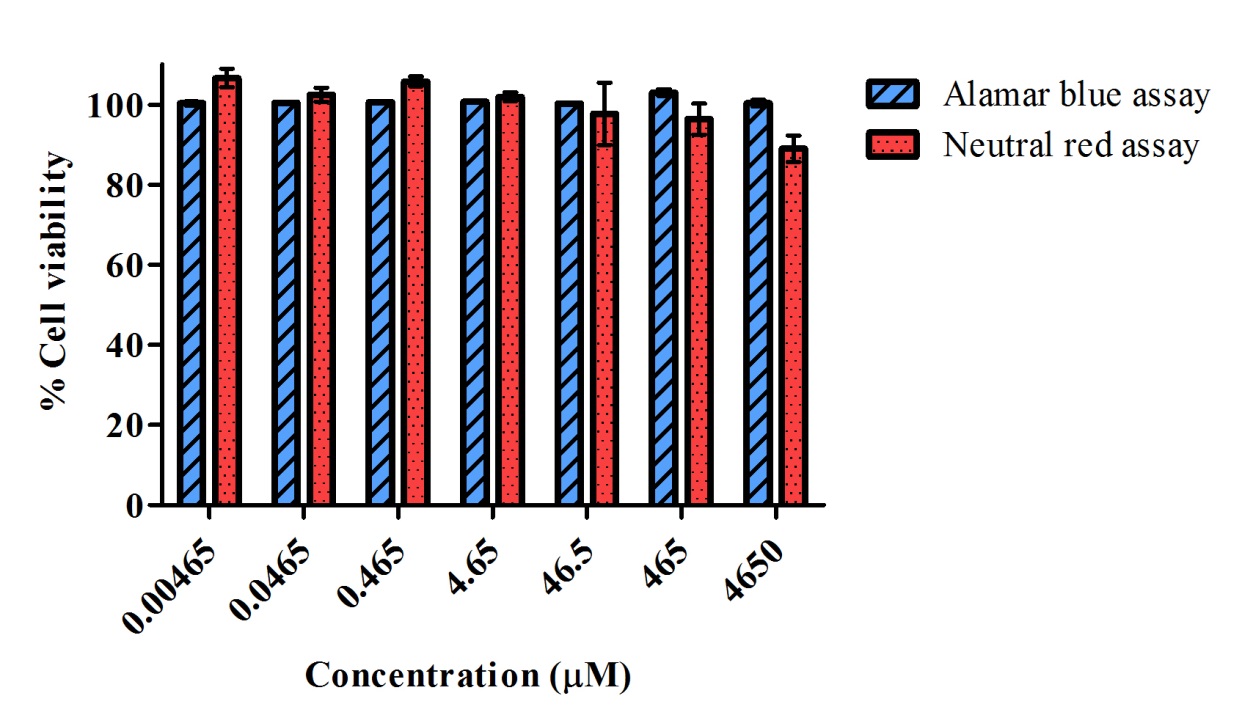


**Fig.S2: Tolerability of HaCaT keratinocytes when treated with (0.005-4,650 μM) of α-T diluted in 0.5% ethanol, 95% Dulbecco's modified eagle's cell culture medium (with FBS) at 37°C for 24 hours.** The α-T was well tolerated by the HaCaT keratinocytes when assessed using both the Alamar blue^®^ (blue columns) and neutral red (red columns) cell viability assays. All the concentrations show viability ≥ 90%. Data represents mean ± standard deviation, (n=3).


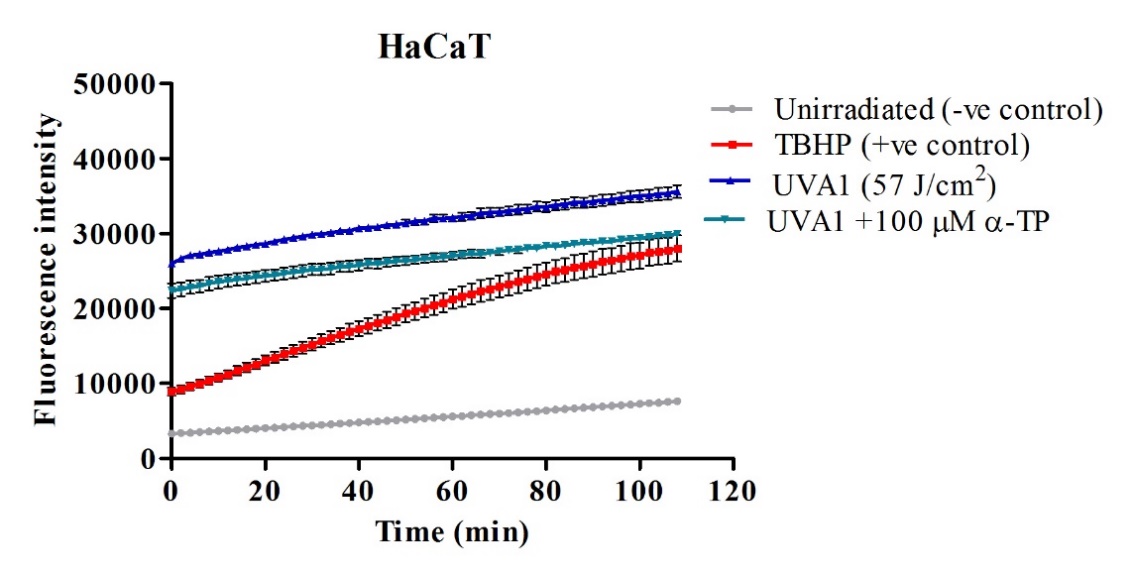


**
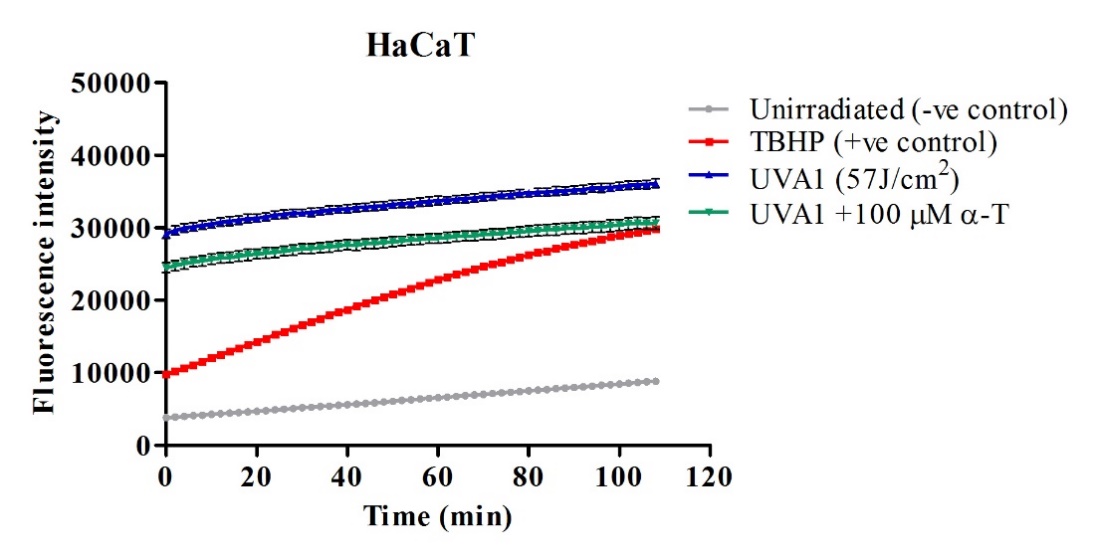
**

**Fig.S3: Pre-treatment studies on HaCaT keratinocytes. The generation of ROS in HaCaT keratinocytes at 2 min intervals after UVA1 irradiation over 1.8-2 h.** HaCaT were pretreated with 100 µM of α-TP and α-T for 24 h and subsequently re-incubated with 20 µM DCFDA for 45 min and then immediately washed with PBS and exposed to a UVA1 dose of 57 J/cm^2^. The TBHP was added into the reserved positive control wells at at concentration of 250 µM. Data represent mean ± SD (n=3).

**Table S3: The generation of ROS in HaCaT keratinocytes pre-treated with antioxidants**

| Condition | \| \| Mean (n=3) difference (a.u.) over 2 h  compared to unirradiated control \| \| --- \| \| \| --- \| --- \| | | |
| --- | --- | --- | --- | --- | --- |
|  | Positive control (TBHP) | 385 nm UVA1 (57 J/cm^2^) | 385 nm UVA1 (57 J/cm^2^) + pre-treatment (100 µM) |
| HaCaT + pre-αTP | -8,414,000 | -14,220,000 | -10,800,000 |
| HaCaT + pre-α-T | -7,685,000 | -13,020,000 | -9,908,000 |

**
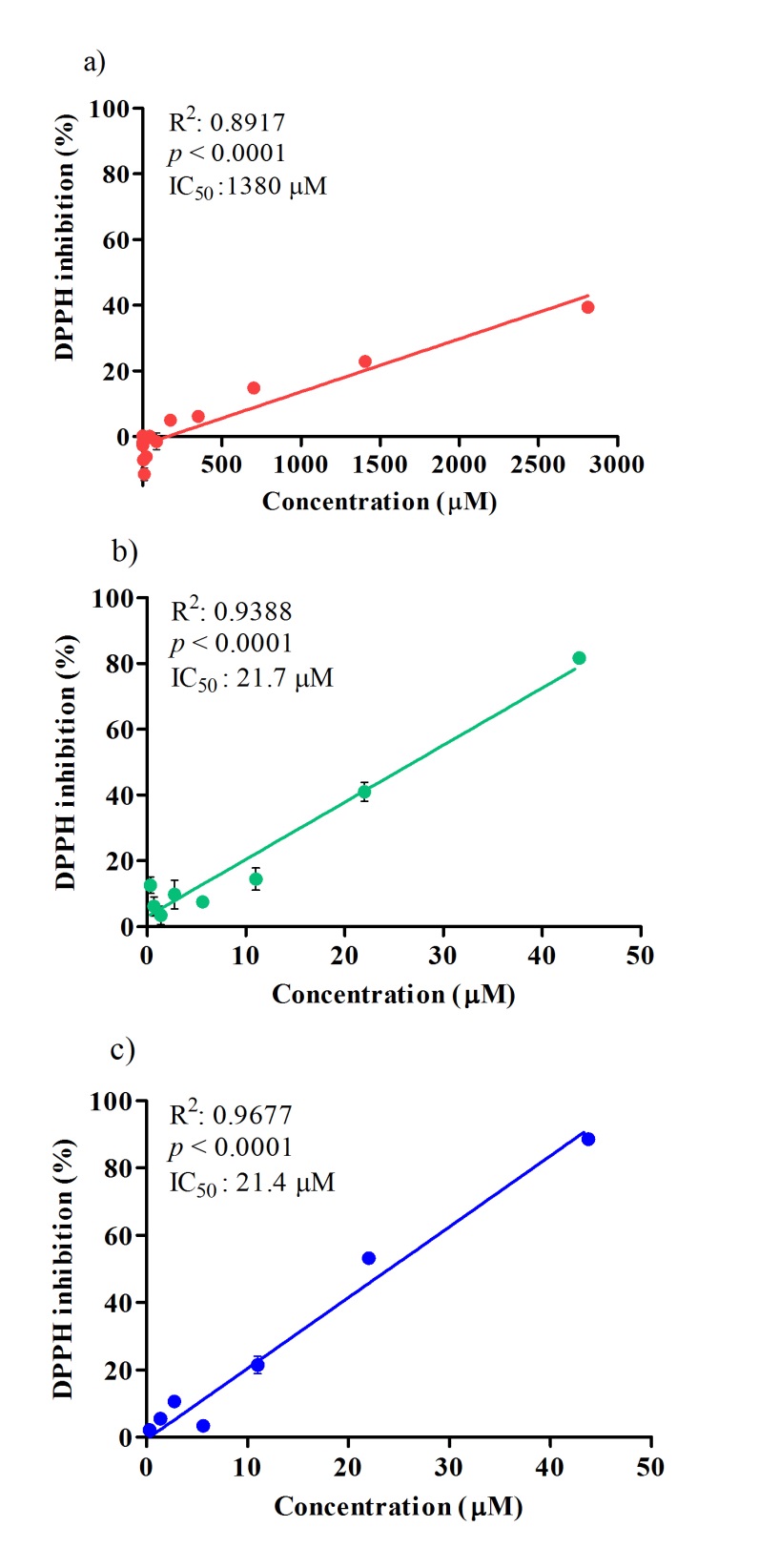
**

**Fig.S4: The DPPH radical scavenging/inhibition activity of α-TP (a), L-ascorbic acid (b), and α-T (c). All tested antioxidants show an ability to quench the DPPH radical as a measure of radical scavenging activity in a concentration-dependant manner.** All compounds demonstrated significant activity (p < 0.0001, linear regression analysis). The standard deviation was too small to be seen (n=3).
